# LRRK2 kinase inhibition prevents pathological microglial phagocytosis in response to HIV-1 Tat protein

**DOI:** 10.1186/1742-2094-9-261

**Published:** 2012-11-29

**Authors:** Daniel F Marker, Jenna M Puccini, Taryn E Mockus, Justin Barbieri, Shao-Ming Lu, Harris A Gelbard

**Affiliations:** 1 Center for Neural Development and Disease, Department of Neurology, Child Neurology Division, University of Rochester, Rochester, NY, USA

**Keywords:** Phagocytosis, Microglia, HIV-1, Tat, Leucine-rich repeat kinase 2 (LRRK2), AnnexinV, Parkinson’s disease, Brain-specific angiogenesis inhibitor 1 (BAI1)

## Abstract

**Background:**

Human Immunodeficiency Virus-1 (HIV-1) associated neurocognitive disorders (HANDs) are accompanied by significant morbidity, which persists despite the use of combined antiretroviral therapy (cART). While activated microglia play a role in pathogenesis, changes in their immune effector functions, including phagocytosis and proinflammatory signaling pathways, are not well understood. We have identified leucine-rich repeat kinase 2 (LRRK2) as a novel regulator of microglial phagocytosis and activation in an *in vitro* model of HANDs, and hypothesize that LRRK2 kinase inhibition will attenuate microglial activation during HANDs.

**Methods:**

We treated BV-2 immortalized mouse microglia cells with the HIV-1 *trans* activator of transcription (Tat) protein in the absence or presence of LRRK2 kinase inhibitor (LRRK2i). We used Western blot, qRT-PCR, immunocytochemistry and latex bead engulfment assays to analyze LRRK2 protein levels, proinflammatory cytokine and phagocytosis receptor expression, LRRK2 cellular distribution and phagocytosis, respectively. Finally, we utilized *ex vivo* microfluidic chambers containing primary hippocampal neurons and BV-2 microglia cells to investigate microglial phagocytosis of neuronal axons.

**Results:**

We found that Tat-treatment of BV-2 cells induced kinase activity associated phosphorylation of serine 935 on LRRK2 and caused the formation of cytoplasmic LRRK2 inclusions. LRRK2i decreased Tat-induced phosphorylation of serine 935 on LRRK2 and inhibited the formation of Tat-induced cytoplasmic LRRK2 inclusions. LRRK2i also decreased Tat-induced process extension in BV-2 cells. Furthermore, LRRK2i attenuated Tat-induced cytokine expression and latex bead engulfment. We examined relevant cellular targets in microfluidic chambers and found that Tat-treated BV-2 microglia cells cleared axonal arbor and engulfed neuronal elements, whereas saline treated controls did not. LRRK2i was found to protect axons in the presence of Tat-activated microglia, as well as AnnexinV, a phosphatidylserine-binding protein. In addition, LRRK2i decreased brain-specific angiogenesis inhibitor 1 (BAI1) receptor expression on BV-2 cells after Tat-treatment, a key receptor in phosphatidylserine-mediated phagocytosis.

**Conclusion:**

Taken together, these results implicate LRRK2 as a key player in microglial inflammation and, in particular, in the phagocytosis of neuronal elements. These studies show that LRRK2 kinase inhibition may prove an effective therapeutic strategy for HANDs, as well as other neuroinflammatory conditions.

## Background

Human Immunodeficiency Virus-1 (HIV-1) associated neurocognitive disorders (HANDs) are a serious and growing cause of disability despite the use of combination antiretroviral therapy (cART) [1]. HIV-1 invades the central nervous system (CNS) early after infection and targets invading peripheral macrophage and resident microglia, which serve as a viral reservoir [2,3]. While cART suppresses viral replication throughout the host, early viral proteins are still produced in the CNS due to the presence of integrated proviral DNA [4]. In particular, the HIV-1 *trans* activator of transcription (Tat) protein is produced within the CNS despite administration of cART [5,6]. The HIV-1 Tat protein has been found to mediate damage in the CNS by upregulating chemotactic gradients that favor monocyte recruitment with accompanying neurotoxicity [7]. Furthermore, a single dose of Tat in the murine CNS can provide a model for the neuroinflammation, persistent synaptic damage and neurodegeneration associated with HANDs [8].

Leucine-rich repeat kinase 2 (LRRK2) is a 286 kDa signaling protein that has many domains, including a GTPase, a mitogen-activated protein kinase kinase kinase (MAPKKK) and a WD-40 domain [9]. Several of the LRRK2 domains are phosphorylated through both autophosphorylation and constitutive phosphorylation [10]. In particular, phosphorylation of serine 935 (pS935) has been linked to kinase activity in LRRK2 [11], where LRRK2 kinase inhibition has been shown to decrease pS935 in HEK 293 cells [12]. The commercially available LRRK2 kinase inhibitor used in this study is highly specific for LRRK2, as it was found to inhibit only 12 out of 442 kinases based on kinase-binding and biochemical assays [12].

Mutations in LRRK2 have been found to modify susceptibility to several diseases with inflammatory components, including Parkinson’s disease (PD), Crohn’s disease (CD) and leprosy [13-15]. LRRK2 is highly expressed in immune cells, including monocytes, B-cells and T-cells, and this expression has been shown to increase after both lipopolysaccharide (LPS) and lentiviral particle treatment in macrophages [16]. Paradoxically, LRRK2 deficiency exacerbates experimentally induced colitis in mice [17], suggesting a phenotypic role for LRRK2 in CD. Conversely, LRRK2 knockout microglia exhibit attenuated microglial inflammation after LPS exposure, in which microglial activation has been implicated in modulating PD [18,19]. Moreover, LRRK2 has been found to increase nuclear factor-kappa beta (NF-κβ) activity in both CD and PD models [20,21]. Thus, LRRK2 plays an important role in inflammation that may have opposing effects based on the unique microenvironment and signaling pathways associated with the given disorder [22].

LRRK2 is a compelling target in understanding neurodegeneration, as mutations in LRRK2 are the most common single gene cause of PD and are found in both familial and sporadic cases of disease [23,24]. PD is a neurodegenerative disorder that is characterized by a loss of dopaminergic neurons in the substantia nigra (SN)*.* The PD-associated mutation LRRK2(G2019S), which causes an increase in LRRK2 kinase activity, has been shown to cause dendritic degeneration and dopaminergic neuronal loss in LRRK2(G2019S) transgenic mice [25]. These animals also exhibited impaired adult neurogenesis and neurite outgrowth [26]. However, LRRK2 is not strongly expressed in the SN [27] and LRRK2 knockout mice were found to have abnormalities in protein processing in the kidneys and not in the brain [28]. Therefore, recent studies have focused on the role of LRRK2 in microglial inflammation, as the SN has the highest density of microglia in the CNS [29].

Recent studies have implicated LRRK2 in neuroinflammation and microglial inflammatory responses [18,21,30]. The PD-associated mutation, LRRK2(R1441G), which also alters LRRK2 kinase activity, has been shown to increase pro-inflammatory cytokine release in microglia, which was found to induce neurotoxicity [30]. Another publication by Moehle *et al.* has implicated LRRK2 in microglial inflammatory responses after exposure to LPS [18]. These results included inhibition of LPS-induced tumor necrosis factor-α (TNF-α) release, chemotaxis and microglial process extension after LRRK2 knockdown. A similar study by Kim *et al.* indicated that LRRK2 regulates LPS-induced microglial activation by altering NF-κβ, p38 and Jun N-terminal kinase (JNK) signaling [21].

As the pathogenicity of LRRK2 activation in microglia during HANDs remains unexplored, and given the neuropathogenic overlaps between HANDs and PD, we investigated the role of LRRK2 in a novel *in vitro* model of HANDs. Phagocytic engulfment has not been studied in relation to LRRK2, even though LRRK2 has been linked to Rac-1, a small Rho GTPase that plays a role in actin remodeling during phagocytosis [31]. Furthermore, the use of microfluidic chambers utilizes a highly novel and physiologically relevant technique to study this phenomenon. As the effects of Tat have been shown to be reversible [32], preventing pro-inflammatory cytokine release and increased phagocytosis during HANDs may be neuroprotective. This has led us to hypothesize that HIV-1 Tat increases microglial production of pro-inflammatory cytokines and phagocytosis of synaptic elements in a LRRK2-dependent manner.

## Methods

### Cell lines and reagents

We maintained the BV-2 mouse microglia cell line (kind gift of Dr. Sanjay B. Maggirwar) in 10% fetal bovine serum (FBS) (Atlas, Fort Collins, CO, USA, F-0500-A) in (D)MEM (Cellgro, Manassas, VA, USA 15-013-CV) with 2 mM GlutaMax (Gibco, Grand Island, NY, USA, 35050–061) and penicillin-streptomycin (Gibco, 15140–122) at 37°C at 5% CO_2_. We dissected 18-day embryonic rat pups to obtain hippocampal neurons for our microfluidic co-culture experiments. We plated and maintained primary hippocampal neurons in Neurobasal media (Gibco, 21103–049) supplemented with 5% FBS, B27 supplement (Gibco, 17504–044), 2 mM GlutaMax, and 50uM L-glutamic acid (Sigma, St. Louis, MO, USA, G5889-100G). We resuspended and plated BV-2 cells in the same supplemented Neurobasal media for all co-culture experiments. We obtained full length HIV-1 Tat_101_ from Philip Ray (University of Kentucky) that we used for all experiments involving Tat exposure. We purchased the small molecule LRRK2 kinase inhibitor LRRK2-IN-1 (LRRK2i) from Millipore (Billerica, MA, USA, 438193, 1 μM working concentration) and purified AnnexinV from eBioscience (San Diego, CA, USA BMS306, working concentration 1 μg/ml). Unless otherwise noted, we plated BV-2 cells in poly-D-lysine (Sigma, P1149-100 mg) coated 12-well plates or on glass coverslips without penicillin-streptomycin overnight and treated as indicated with 1 μg/ml Tat_101_, saline vehicle control, dimethyl sulfoxide (DMSO) vehicle control, or 1 μM LRRK2i for 12 hours, as indicated by our preliminary time-course experiments (data not shown). For BV-2 cell counts, we used a hemocytometer with 0.4% trypan blue stain (Gibco, 15250).

### Western blotting

We harvested cells by scraping into 1X PBS and centrifuging at 7,000 rpm for 1 minute. We resuspended the pellet in cell lysis buffer with protease inhibitors and lysed it on ice with periodic vortexing for 30 minutes. We normalized protein concentration by Bradford assay (BioRad, Hercules, CA, USA 500–0113, 500–0114, 500–0115). We mixed 10 μg of sample with loading dye, heated it at 70°C for 5 minutes, and ran it on a 4% to 15% SDS-PAGE gel (BioRad, 456–1086) at 100 V for 1 hour. We transferred the gel onto a polyvinylidene difluoride (PVDF) membrane at 100 V for 1 hour on ice. We blocked membranes in 5% milk in 1X Tris-buffered saline (TBS) for 1 hour at room temperature with shaking. We washed membranes 3 times in 1X Tris-buffered saline with 0.1% Triton-X 100 (TBST) . We applied primary antibodies overnight at 4°C with shaking in 5% milk in 1X TBST at the following concentrations; rabbit anti-LRRK2 (1:1000) (Epitomics, Burlingame, CA, USA, 3514–1), rabbit anti-pS935-LRRK2 (1:1000) (Epitomics, 5099–1), β-actin (1:500) (Santa Cruz, Santa Cruz, CA, USA, sc-47778) . We washed membranes 3 times in 1X TBST. We applied horseradish peroxidase (HRP)-conjugated secondary antibody (GE Healthcare, Pittsburgh, PA, USA ) at a concentration of 1:5,000 in 5% milk in 1X TBST for 1 hour at room temperature with shaking. We washed membranes and applied enhanced chemiluminescence (ECL) substrate (Pierce, Rockford, IL, USA, 32106) for 1 minute. We exposed and developed membranes on film (Kodak, Rochester, NY, USA, 111–1681).

### qRT-PCR

We isolated RNA from BV-2 cells using the RNeasy RNA isolation kit (Qiagen, Valencia, CA, USA, 74104). We synthesized cDNA from 0.5 μg of total RNA using the SuperScript III First-Strand Synthesis System (Invitrogen, Carlsbad, CA, USA, 18080–051). We used the TaqMan Universal PCR Master Mix (Invitrogen, 4304437) with Invitrogen TaqMan Gene Expression Assay primers and probed for TNF-α (Mm00443258_m1), IL-6 (Mm00446190_m1), MCP-1 (Mm00441242_m1), IL-10 (Mm00439614_m1), IL-4 (Mm00445259_m1), BAI1 (Mm01195143_m1), Tim4 (Mm00724709_m1) and 18S ribosomal RNA (Mm03928990_g1) as an internal control. We ran and measured the samples on an Applied Biosystems (Carlsbad, CA, USA) StepOnePlus real time PCR machine. We analyzed relative target gene levels by comparing the fold change of delta-delta threshold cycle to the control after normalization to 18S ribosomal RNA.

### Immunocytochemistry

We adapted our immunocytochemistry (ICC) protocol from Glynn *et al.*[33]. Briefly, we fixed the cells in a solution of 4% paraformaldehyde, 4% sucrose in 1x PBS at 4C for 10 minutes. We then treated the fixed cells with 100 mM glycine in 1x PBS and washed in 1x PBS for 5 minutes. We prepared the primary antibodies in 3% BSA (Sigma, A3294-50 G) in 1x PBS at the following concentrations: rat anti CD11b 1:200 (Serotech, MCA711), mouse anti Tau5 1:500 (Calbiochem, 577801), rabbit anti Synapsin-1 1:500 (Cell Signaling, 5297S), and rabbit anti LRRK2 1:500 (Novus, St. Charles, MO, USA, NB300-268). We incubated in the fixed cells in the primary antibody solution for 1 hour, then washed 4 times with 3% BSA, 1x PBS for five minutes apiece. We prepared alexa fluor secondary antibodies (Molecular Probes, Eugene, OR, USA) in 3% BSA, 1x PBS at 1:500, and incubated the cells in secondary antibody for 30 minutes. We then washed the cells 4 times in 1x PBS for five minutes apiece, before mounting them on glass slides using the Prolong Gold with 4',6-diamidino-2-phenylindole (DAPI) mounting agent (Life Technologies, Carlsbad, CA, USA, P36935).

### Latex bead-based phagocytosis assays

We exposed experimentally-treated BV-2 cells to 0.8 μm average diameter deep blue dyed uncharged latex beads (Sigma, L1398) for 1.5 hours, after which we washed, scraped, and sonicated the cell/bead suspension. We measured the absorbance of the resulting solution on a visible light spectrophotometer at 595 nm to obtain a quantitative readout for bead phagocytosis. We also exposed BV-2 cells experimentally treated as indicated for 6 hours with 1 μm average diameter fluorescent, carboxylated latex beads (Invitrogen, F8816) for 45 minutes. We washed and fixed the cells as in our ICC protocol to obtain a qualitative readout.

### Microfluidic chamber system

We fabricated photoresist molds and polydimethylsiloxane (PDMS) microfluidic chambers as described by Park *et al.*[34]. We plasma-cleaned 22 mm square #1.5 coverglass (Corning, Tewksbury, MA, USA, 2870–22) and submerged it in 50 μg/ml poly-d-lysine at 37°C overnight. We thoroughly washed and dried the glass and assembled the chambers by placing the molded PDMS on top of the glass and allowing it to seal. We then filled the chamber with media in the order described by Park. After removing the media, we added 20 μl of a 3.5 × 10^6^ hippocampal cells/ml cell suspension to the upper left well of the chamber. We allowed the cells to attach for 15 minutes, and then filled the chamber with media as described by Park. We observed axons crossing the 400 μm barrier by 3 days *in vitro* (DIV), with extensive neuritic networks forming by 7 DIV.

All of the chambers we used for the microfluidic experiments were 7 DIV. We imaged the chambers before treatment to obtain baseline axonal images and measurements. We then plated the experimentally treated BV-2 cells in the axon compartment by carefully removing the media from the axon wells and adding 20 μl of a 3 × 10^6^ BV-2 cells/ml cell suspension to one of the axon compartment wells. We allowed the cells to attach and then filled the chamber with media. We utilized a media pressure gradient to assure that none of the soluble experimental manipulations diffused to the cell body compartment. We cultured the chambers for a further 14 hours and then obtained post treatment brightfield images. We then deconstructed the chambers and fixed the cells with 4% paraformaldehyde (Sigma, 441244), 4% sucrose (EM Science, Gibbstown, NJ, USA, 8510) in 1x PBS for future immunocytochemistry. Based on pilot experiments with MAP-2 and Tau5 (see below), by 7 DIV, no MAP-2 positive processes were identified that traversed the entire 400 μm barrier (data not shown), suggesting that only axonal processes and growth cones were present in compartment 2.

### Imaging and analysis

For our fluorescent fixed cell ICC, we imaged slides on an Olympus BX-51 upright microscope equipped with Qioptic Optigrid optical sectioning hardware (Princeton, NJ, USA). We imaged the fixed cells with a 40x 0.8NA air objective with a 0.2 um z-step or using a 20x 0.58NA air objective with a 0.5 um z-step. We captured images using a Hamamatsu ORCA-ER camera controlled by Perkin Elmer Volocity software, and created the representative images of the three-dimensional stacks using a z-projection in the same software. For our live cell bright-field images, we imaged the microfluidic chambers on an Olympus IX70 inverted microscope under 10x magnification. We captured the images using a PCO.edge camera controlled by the NIH image capturing software Micromanager [35]. We processed the images using NIH ImageJ [36] image analysis software, with the addition of the NeuronJ Java plugin for axon length analysis ( http://www.imagescience.org/meijering/software/neuronj/) [37].

## Results

### LRRK2 expression in BV-2 microglia cells

**Figure 1:**
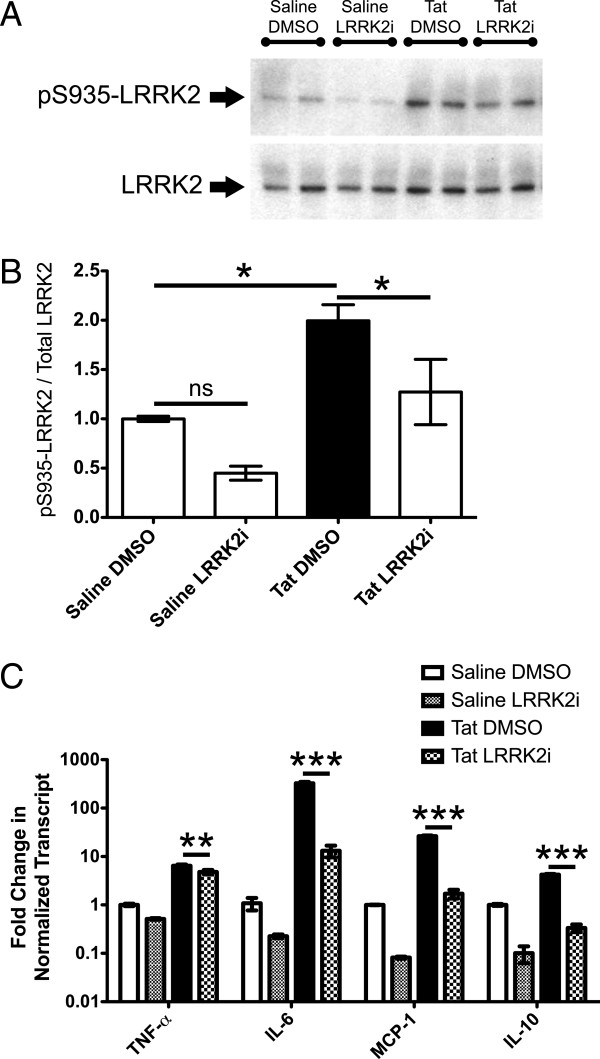
**LRRK2 kinase inhibition blocks Tat-induced S935 phosphorylation and inflammatory cytokine expression in BV-2 cells.** (**A**) Western blot depicting pS935-LRRK2 and total LRRK2 protein levels 12 hours after saline or Tat-treatment ± LRRK2 kinase inhibition (LRRK2i). (**B**) Tat treatment significantly increased pS935-LRRK2, which was attenuated by LRRK2i. (**C**) We measured TNF-α, IL-6, MCP-1 and IL-10 mRNA levels by qRT-PCR 12 hours post saline or Tat treatment ± LRRK2i. LRRK2i attenuated Tat-induced TNF-α, IL-6, MCP-1 and IL-10 expression for all groups (*P <0.05, ***P <0.001, one way ANOVA, Newman-Keuls post-test). ANOVA, analysis of variance; LRRK2, leucine-rich repeat kinase2; pS935, phosphorylation of serine 935; Tat, trans activator of transcription.

In order to study LRRK2 activity in microglia, we utilized the BV-2 mouse microglia cell line. BV-2 microglia cells are biochemically similar to microglial cells found in the murine CNS and grow robustly in culture [38]. We found that Tat-treatment of BV-2 cells induced the kinase activity associated pS935 on LRRK2, which was decreased by inhibition of LRRK2i (Figure 1A, B).

### LRRK2 kinase inhibition attenuates Tat-induced microglial cytokine expression

To examine microglial activation in response to HIV-1 Tat protein, we analyzed proinflammatory cytokine expression through qRT-PCR. TNF-α, IL-6 and MCP-1, also known as CCL2, were significantly increased after Tat treatment and attenuated through LRRK2i (Figure 1C). Tat treatment did not induce IL-4 expression in BV-2 cells (data not shown). These particular cytokines were analyzed due to their increased expression in the CNS during HANDs and contribution to neurotoxicity [39,40]. Additionally, MCP-1 is a potent inducer of peripheral monocyte infiltration into the CNS during neuroinflammation, and also causes migration of resident microglia [41,42].

### Altered LRRK2 cytoplasmic distribution and microglial process length after HIV-1 Tat exposure

**Figure 2:**
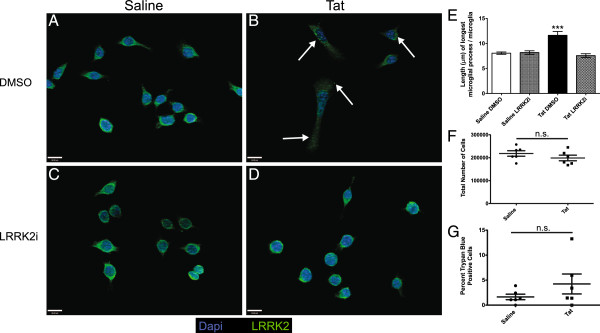
**LRRK2 kinase inhibition prevents the formation of Tat-induced LRRK2 positive inclusions and normalizes process length in BV-2 cells.** We treated BV-2 cells for 12 hours with saline or Tat ± LRRK2i and immunostained for LRRK2 (green). (**A**) Saline-only treated cells contain diffuse cytoplasmic LRRK2 expression. (**B**) Tat-treated cells contain brightly staining LRRK2 positive inclusions (white arrows). (**C**, **D**) BV-2 cells treated with LRRK2i and either saline or Tat do not contain the inclusions seen in the Tat alone treated group. (**E**) Graph showing average length of longest microglia process in various experimental conditions. We averaged the longest processes from 100 microglia in monoculture from each condition. Tat-treated microglia had significantly longer processes than saline-treated microglia. LRRK2i normalized the microglia process length in the presence of Tat and had no effect in the presence of saline (***P <0.001, one way ANOVA, Newman-Keuls post-test). In order to ensure that the changes in BV-2 cell morphology were not accompanied by changes in cell proliferation or cell death, we performed total cell counts and trypan blue staining after 12 hours of Tat treatment. We found no statistical difference in total cell number (**F**) or cell death (**G**) after 12 hours of Tat exposure (*P* = 0.28 and *P* = 0.24 respectively, Student’s t-test). ANOVA, analysis of variance; LRRK2, leucine-rich repeat kinase 2; LRRK2i, LRRK2 inhibitor; Tat, trans activator of transcription.

We then examined the effect of Tat and LRRK2i on LRRK2 distribution within BV-2 cells and on BV-2 cell morphology. ICC against LRRK2 revealed diffuse staining throughout the cytoplasm in saline control cells (Figure 2A). When the BV-2 cells are exposed to Tat, LRRK2 expression appears to be concentrated in small 1 μm inclusions found throughout the cytoplasm (Figure 2B). The addition of LRRK2i to the saline treated condition had no effect on LRRK2 distribution (Figure 2C), while cytoplasmic inclusions were no longer observed in the Tat-treated condition after the addition of LRRK2i (Figure 2D). BV-2 cells morphologically responded to Tat exposure by extending processes (Figure 2B), as compared to their normal amoeboid morphology (Figure 2A). While Tat increased microglia process length, LRRK2i significantly decreased process length to that of saline controls (Figure 2D, E). These results expand on previously published data showing that LRRK2i attenuates LPS-induced microglia process extension [18]. Furthermore, BV-2 cell counts indicated that Tat-treatment did not significantly alter proliferation (Figure 2F) or cell death (Figure 2G) over the experimental time course.

### Tat-induced microglial phagocytosis of uncharged and carboxylated latex beads is reduced by LRRK2 kinase inhibition

**Figure 3:**
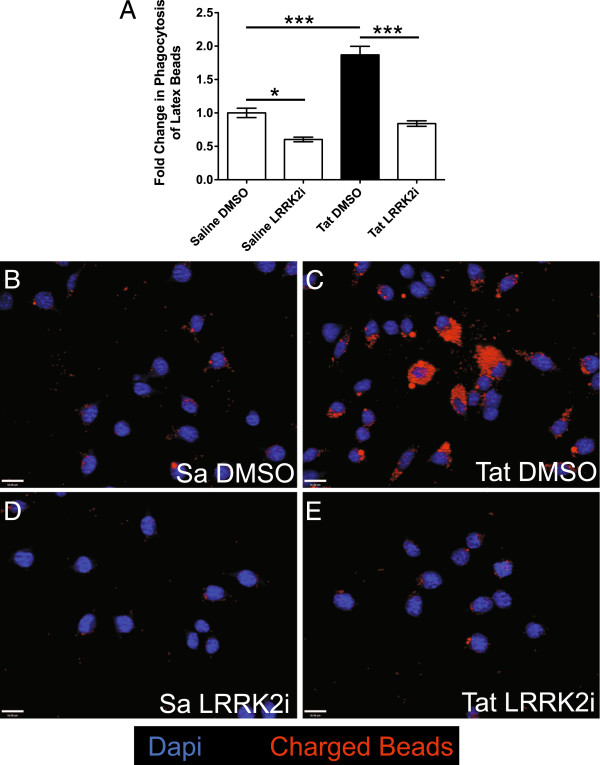
**LRRK2 kinase inhibition attenuates Tat-induced microglial phagocytosis of uncharged and carboxylated latex beads.** We treated BV-2 cells with saline or Tat for 12 hours ± LRRK2i and then exposed the cultures to uncharged latex beads for 1.5 hours. (**A**) LRRK2i significantly decreased Tat-induced phagocytosis of uncharged latex beads (n = 3 per condition). We then treated BV-2 cells with Tat for 6 hours and exposed them to carboxylated, fluorescent latex beads for 45 minutes. Cells treated with Tat (**C**) contain many more latex beads (red) than saline treated cells (**B**). Treatment with LRRK2i in both the saline (**D**) and Tat (**E**) conditions decreased the number of engulfed beads (*P <0.05, ***P <0.001, one way ANOVA, Newman-Keuls post-test). ANOVA, analysis of variance; LRRK2, leucine-rich repeat kinase 2; LRRK2i, leucine-rich repeat kinase 2 inhibitor; Tat, trans activator of transcription.

In order to ascertain phagocytic capacity within Tat-treated BV-2 cells, we utilized both uncharged and carboxylated latex bead engulfment assays. We found that Tat treatment significantly increased phagocytosis of uncharged, blue dyed latex beads by BV-2 cells, as compared to saline treated controls using a quantitative read-out (Figure 3A). Co-treatment with LRRK2i decreased Tat-induced phagocytosis of uncharged latex beads and also decreased basal levels of phagocytosis in saline controls treated with LRRK2i (Figure 3A). We also exposed BV-2 cells to fluorescent, carboxylated beads, in which the negative charge on the bead mimics phosphatidylserine exposure on apoptotic cells [43-45]. We found that Tat-treated BV-2 cells engulfed more beads (Figure 3C, red signal) as compared to saline (Figure 3B) or Tat co-treatment with LRRK2i (Figure 3E). Furthermore, LRRK2i also decreased carboxylated bead engulfment in saline controls (Figure 3D), similar to results seen with uncharged beads (Figure 3A). These results indicate that treatment with LRRK2i is an effective way to reduce HIV-1 Tat-induced microglial phagocytosis, which may be partially mediated by phosphatidylserine exposure on apoptotic cell targets.

### Primary axonal clearance by Tat-treated microglia in microfluidic chambers is reduced by LRRK2 kinase inhibition

**Figure 4:**
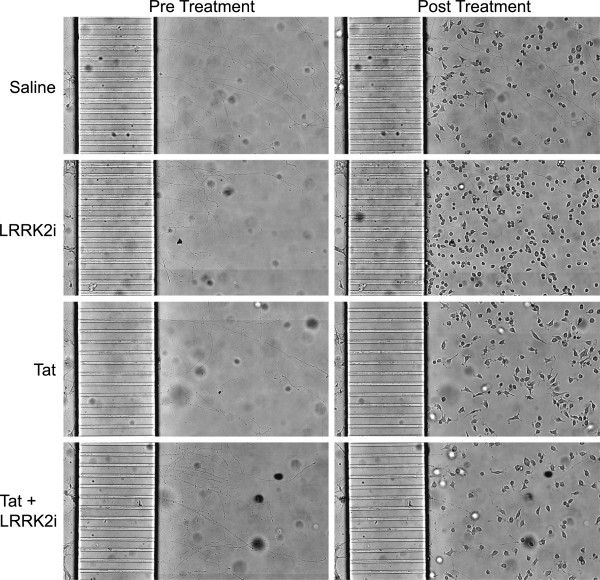
**LRRK2 kinase inhibition protects primary axons from Tat-induced microglial phagocytosis.** We co-cultured BV-2 cells along with various experimental conditions in the axonal compartment of established rat hippocampal primary neuron microfluidic chambers for 18 hours. We imaged the axonal field before and after treatment to determine the effects of the experimentally treated microglia on the neuronal structures. The figure shows representative images of axons before and after co-culture with experimentally treated BV-2 cells. Axons exposed to saline or LRRK2i treated BV-2 cells remain wholly intact, while axons exposed to Tat-treated BV-2 cells are largely destroyed. The lack of axonal debris indicates the presence of microglial phagocytosis. The addition of LRRK2i partially protects the axon field from the Tat treated BV-2 cells. LRRK2i, leucine-rich repeat kinase 2 inhibitor; Tat, trans activator of transcription.

As our laboratory has previously shown that microglia engulf neuronal components after exposure to HIV-1 Tat in the murine CNS [8], we used *in vitro* microfluidic chambers to model microglial-axonal interactions. We grew primary hippocampal neurons in microfluidic chambers that allow for physical and fluidic isolation of axons from soma. This separation allows us to study potential microglial phagocytosis of neuronal processes in the absence of neuronal cell bodies. We grew the neurons in the chambers for seven days, by which time they had developed an extensive axonal arbor (Figure 4, pre treatment). We then plated BV-2 cells in the presence of various experimental conditions in the axonal compartment, and co-cultured the cells for a further 18 hours (Figure 4, post treatment).

**Figure 5:**
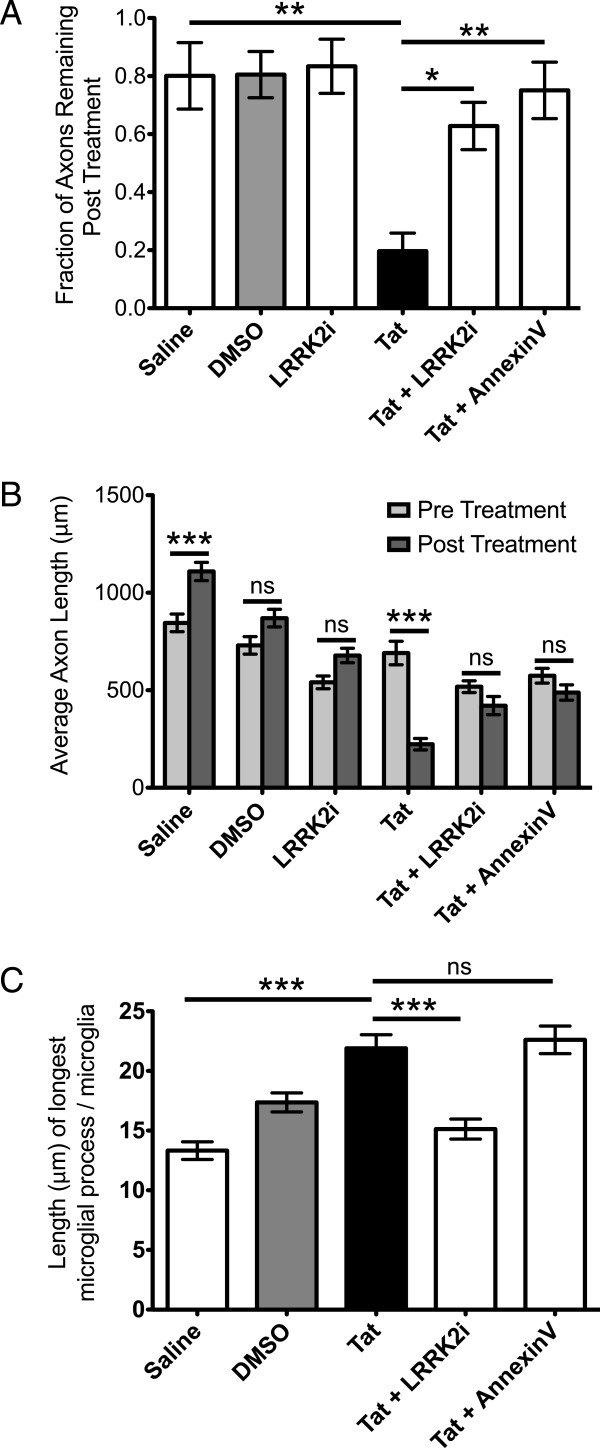
**Quantification of the effects of LRRK2i and AnnexinV on Tat induced neuronal axon elimination, neuronal axon length, and microglia process length.** (**A**) Approximately 80% of axons remained in microfluidic chambers after exposure to saline, DMSO, or LRRK2i alone treated BV-2 cells, where only 20% of axons remained after the addition of Tat treated BV-2 cells. The addition of LRRK2i or AnnexinV to Tat treated BV-2 cells significantly increased survival rate to 63% and 75%, respectively (n = 3 chambers per condition, *P <0.05, **P <0.01, one way ANOVA, Newman-Keuls post-test). (**B**) Axons exposed to Tat-treated BV-2 cells are significantly shorter post treatment. Axons exposed to either DMSO, LRRK2i alone, Tat and LRRK2i, or Tat and AnnexinV treated BV-2 cells exhibited no significant change. Axons exposed to saline treated BV-2 cells were significantly longer post treatment (n = a minimum of 30 axons per experimental condition, ***P <0.001, two way ANOVA, Bonferroni post test). (**C**) Tat treated microglia had significantly longer processes than saline treated microglia. LRRK2 kinase inhibition normalized the microglia process length in the presence of Tat. BV-2 cells exposed to Tat and AnnexinV were not significantly different from Tat alone (n = longest process of 100 microglia in co-culture, *P <0.05, **P <0.01, ***P <0.001, one way ANOVA, Newman-Keuls post-test). ANOVA, analysis of variance; DMSO dimethyl sulfoxide; LRRK2i, leucine-rich repeat kinase 2 inhibitor; Tat, trans activator of transcription.

**Figure 6:**
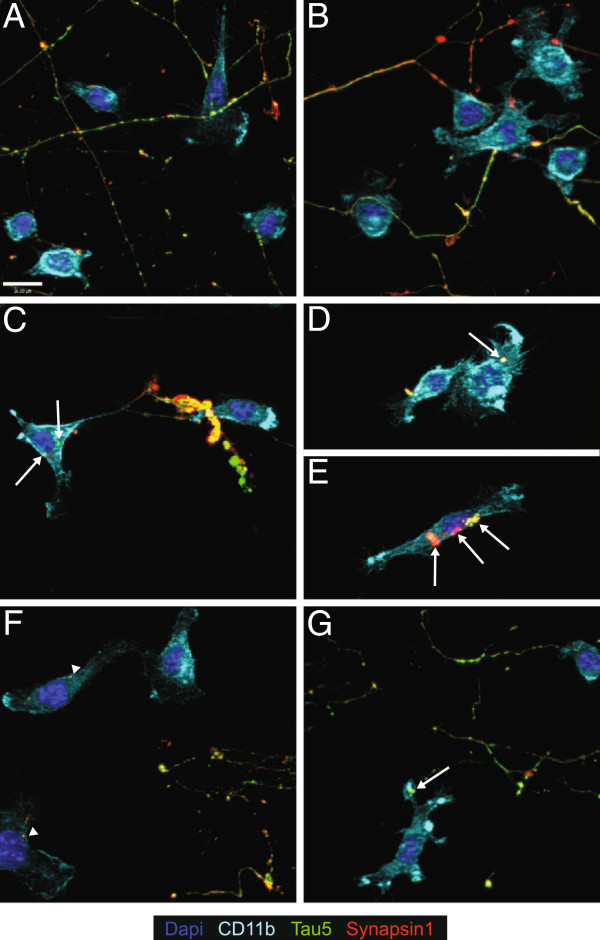
**Tat-treated BV-2 cells cultured with primary axons contain inclusions of neuronal components that are altered by LRRK2 kinase inhibition.** We immunostained BV-2-neuronal cultures for the axonal cytoskeletal protein Tau 5 (green), the axonal terminal protein Synapsin-1 (red), and the microglial membrane protein CD11b (light blue) after 18 hours of experimental treatment. Saline (**A**) and DMSO (**B**) treated BV-2 cells do not appear to phagocytose any neuronal components. Tat treated BV-2 cells (**C**, **D**, **E**) contain Tau-5 and Synapsin-1 positive inclusions, indicative of axonal phagocytosis (white arrows). Tat exposed BV-2 cells treated with LRRK2i (**F**) contain some neuronal inclusions that are much smaller than Tat exposed alone (white arrowheads), indicating a stunted phagocytic process. Tat exposed BV-2 cells treated with AnnexinV (**G**) contain fewer inclusions than Tat alone, but the inclusions are the same size as the Tat alone treated condition (white arrows). LRRK2i, leucine-rich repeat kinase 2 inhibitor; Tat, trans activator of transcription.

BV-2 cells exposed to saline (1 μl/ml), DMSO (1 μl/ml), or LRRK2i alone (1 μM) did not damage the existing axonal arbor, as indicated by axon persistence (Figures 4, 5A) and growth (Figures 4, 5B). BV-2 cells exposed to Tat (1 μg/ml), on the other hand, completely destroyed the existing axonal arbor and prevented any further axon growth (Figures 4, 5A, 5B). The lack of remaining axonal debris indicated microglial phagocytosis of axons. We confirmed this finding using ICC, with many BV-2 cells in the Tat treated condition containing Tau5 or Synapsin-1 positive inclusions (Figure 6C, D, E). These inclusions were not present in the saline (Figure 6A) or DMSO (Figure 6B) conditions. The addition of LRRK2i (1 μM) to Tat treated BV-2 cells protected the axons from microglial clearance (Figures 4, 5B, 5A). When we examined these cells using ICC, we found the presence of some inclusions that stained positive for neuronal markers, but these inclusions were much smaller and morphologically different from those in the Tat treatment alone (Figure 6F).

### Tat-induced axonal phagocytosis is partially dependent on phosphatidylserine exposure to BV-2 cells

**Figure 7:**
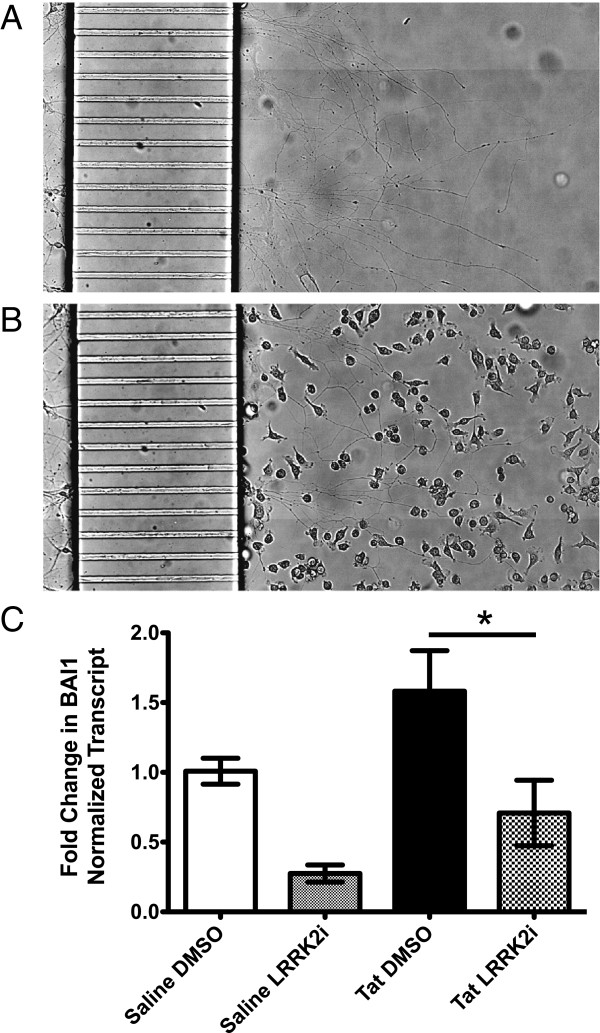
**Tat-induced microglial phagocytosis of axons is partially dependent on phosphatidylserine signaling.** In order to determine if Tat-induced BV-2 cell phagocytosis of axons was phosphatidylserine dependent, we blocked phosphatidylserine exposure with AnnexinV. (**A**) Microfluidic chambers are shown before treatment. (**B**) The addition of AnnexinV protected the axon field from Tat-treated BV-2 cells in microfluidic chambers. (**C**) Using qRT-PCR, we measured the expression of the phosphatidylserine receptor BA1 in monoculture BV-2 cells exposed to Tat or saline for 12 hours ± LRRK2i. Tat treatment increased BA1 expression, although not significantly. LRRK2i significantly decreased BA1 expression in Tat treated BV-2 cells (*P <0.05, one way ANOVA, Newman-Keuls post-test). ANOVA, analysis of variance; LRRK2i, leucine-rich repeat kinase 2 inhibitor; Tat, trans activator of transcription.

To determine if microglial phagocytosis of axons was phosphatidylserine dependent, we co-treated BV-2s with Tat and AnnexinV. AnnexinV (1 μg/ml) protected the axonal field from the Tat treated BV-2 cells (Figures 5A, 5B, 7A-B). BV-2 cells treated with both Tat and AnnexinV did contain neuronal marker positive inclusions similar to those in the Tat only condition (Figure 6G), but at a far smaller rate than Tat alone (data not shown). In order to investigate how LRRK2i may inhibit phagocytosis based on this result, we measured the expression of two phosphatidylserine receptors, BAI1 and TIM4, in BV-2 cells. We did not find any TIM4 expression in BV-2 cells (data not shown). BAI1 expression increased after Tat treatment, although not significantly (Figure 7C). LRRK2i significantly decreased BAI1 expression to control levels in Tat-treated BV-2 cells (Figure 7C). Taken together, these results indicate a role for phosphatidylserine receptor mediated phagocytosis of neuronal components.

### Microglia morphology is altered by Tat treatment and LRRK2i during microfluidic coculture with primary neuronal axons

Finally, we analyzed the morphology of the experimentally treated microglia in the presence of axons. We found that Tat exposed microglia extended processes longer than controls, and that this process is inhibited with the addition of LRRK2i, similar to our findings in monocultures of BV-2 cells (Figure 5C). Surprisingly, the addition of AnnexinV did not inhibit microglia process extension (Figure 5C), indicating that the microglia are still activated despite the absence of neuronal phagocytosis in this condition.

## Discussion

LRRK2 is a large signal transduction protein with a MAPKKK domain. Mutations in LRRK2 are strongly linked to Parkinson’s disease (PD) [9]. The principal finding from our data supports the hypothesis that LRRK2 regulates microglial activation after exposure to HIV-1 Tat, affecting both microglial pro-inflammatory cytokine expression and phagocytosis. This functional change in microglia may account for synaptic damage, which is a cornerstone of the neuropathology observed in HANDs [46].

While it is known that Tat induces the release of the pro-inflammatory cytokine TNF-alpha in microglia, how Tat alters phagocytosis in microglia is not well understood [47]. Several recent publications from the Brown group show that preventing microglia phagocytosis is sufficient to protect neurons exposed to either LPS or micromolar levels of amyloid beta (Aβ) [48-51]. These results indicate a primary role of microglial phagocytosis in certain neurodegenerative and neuroinflammatory conditions. Our *in vitro* microfluidic co-culture system mimics these findings in the context of HANDs. A recent publication indicated that treatment with Tat decreased phagocytosis of Aβ peptide in primary microglia [52], indicating that microglial phagocytosis may be substrate-specific during neuroinflammation. These data add an additional dimension of complexity to microglial mechanisms of neuroinflammation when multiple disorders occur simultaneously within the CNS and highlight the importance of understanding microglial phagocytosis during neurodegeneration.

Several recent studies investigating LRRK2 in LPS-induced microglial activation have shown that LRRK2 knockout and the LRRK2(R1441G) mutation attenuated and augmented microglial inflammation, respectively [18,21,30]. Interestingly, phosphorylation of LRRK2 at S910 and S935 occurs during Toll-like receptor signaling in bone marrow derived macrophages [53]. Our results extend these findings to show that Tat treatment also induces phosphorylation at S935 of LRRK2, suggesting additional loci for Tat to perturb LRRK2 function in infiltrating pro-inflammatory macrophages in the CNS.

Furthermore, LRRK2 has been found to form inclusions when in the presence of LRRK2i in HEK-293 cells [12], lymphoblastoid cells [11], and rat brain [54]. We did not observe LRRK2 inclusions in the presence of LRRK2i in BV-2 cells, which may indicate that this phenomenon is cell-type specific. However, we did observe LRRK2 aggregation after Tat treatment in BV-2 cells, in the form of several 1 μm LRRK2-positive vesicles. Recent publications have linked LRRK2 to the autophagy pathway in macrophages [16], kidney [28] and brain [55-57]. Future studies from our laboratory will focus on the purpose of these inclusions and whether they represent an altered role for LRRK2 and the autophagy pathway within these cells.

There is a distinct possibility that some of the axonal protection we observe with the use of the LRRK2i is provided by LRRK2 kinase inhibition in the axons themselves, and not from the anti-inflammatory effects in the microglia alone. LRRK2 kinase over-activity, specifically due to the LRRK2(G2019S) mutation, has been shown to decrease neurite outgrowth and complexity, in both *in vitro* and *in vivo* models [26,56-59]. It is, therefore, possible that LRRK2 kinase inhibition may impart some protection to established axons, but the effect of LRRK2 kinase over-activity or inhibition in established axons in our paradigms has not yet been directly studied.

Our studies did not attempt to establish a mechanism for how LRRK2 inhibition may prevent microglia phagocytosis. However, the ability of AnnexinV to block axonal phagocytosis and the decrease in BAI1 expression in Tat-treated BV-2 cells after LRRK2i treatment indicates that Tat-induced phagocytosis is, at least in part, phosphatidylserine-dependent. Additionally, while the role of LRRK2 in effecting the neuronal cytoskeleton has been well established [58], a recent publication has implicated LRRK2 more generally in actin cytoskeletal dynamics [60]. Actin reorganization is critical in microglial phagocytosis, providing a potential mechanism for the reduced phagocytosis in the LRRK2i treated BV-2 cells [61]. Furthermore, LRRK2 knockdown has been found to reduce reactive oxygen species production during bacterial engulfment in macrophages [20].

These findings provide the basis for future studies, not only in relation to LRRK2 and phagocytosis, but also to better understand the mechanisms of neuronal phagocytosis by microglia. These phenomena have been implicated in several neurodegenerative and neuroinflammatory diseases, such as PD, Alzheimer’s disease and HANDs [8,50,51]. This work may lead to novel LRRK2-based therapeutic strategies to inhibit microglial inflammation and phagocytosis of the neuronal arbor.

## Conclusions

LRRK2i limits inflammatory cytokine expression and phagocytosis of neuronal components by microglia exposed to HIV-1 Tat. These results implicate LRRK2i as a potential means for developing an adjunctive therapy for HANDs, as well as other neuroinflammatory disorders where LRRK2 may be contributing to pathology.

## Abbreviations

Aβ: Amyloid β; BSA: Bovine serum albumin; cART: Combination antiretroviral therapy; CNS: Central nervous system; (D)MEM: (Dulbecco’s) modified Eagle’s serum; ECL: Enhanced chemiluminescence; FBS: Fetal bovine serum; HANDS: HIV-1 associated neurocognitive disorders; ICC: Immumocytochemistry; IL: Interleukin; LPS: Lipopolysaccharide; LRRK2: Leucine-rich repeat kinase 2; LRRK2i: Leucine-rich repeat kinase 2 inhibitor; MAPKKK: Mitogen-activated protein kinase kinase kinase; NF-κβ: Nuclear factor-kappa beta; PBS: Phosphate-buffered serum; PD: Parkinson’s disease; pS935: Phosphorylation of serine 935; PVDF: Polyvinylidene difluoride; qRT-PCR: Quantitative reverse transcriptase-polymerase chain reaction; Tat: Trans activator of transcription; TNF-α: Tumor necrosis factor α.

## Competing interests

The authors declare that they have no competing interests.

## Authors’ contributions

DFM carried out microfluidic chamber experiments and immunocytochemistry. JMP, TEM and JB carried out qRT-PCR and phagocytosis assay experiments. JMP carried out western blots and immunocytochemistry. DFM, JMP, SML, and HAG participated in data analysis and writing of the manuscript. All authors read and approved the final manuscript.
